# Aggressive Management of Pulmonary Mucormycosis Necrotizing Fasciitis in a Post-Liver Transplant Patient

**DOI:** 10.7759/cureus.78649

**Published:** 2025-02-06

**Authors:** Shivam Chandra, Marc Moza, Ryan Hubbard, Michael Bishop

**Affiliations:** 1 General Surgery, A.T. Still University School of Osteopathic Medicine in Arizona, Mesa, USA; 2 Osteopathic Medicine, A.T. Still University School of Osteopathic Medicine in Arizona, Mesa, USA; 3 Internal Medicine, A.T. Still University School of Osteopathic Medicine in Arizona, Mesa, USA; 4 Cardiovascular and Thoracic Surgery, RUSH University Medical Center, Chicago, USA

**Keywords:** antifungal therapy, bronchoalveolar lavage, eloesser flap procedure, immunocompromised infections, liver transplantation, necrotizing fasciitis, post-transplant infections, pulmonary mucormycosis, surgical debridement

## Abstract

Pulmonary mucormycosis (PM) is a severe fungal infection that predominantly affects immunocompromised and diabetic individuals, and it is associated with a high mortality rate, particularly in cases of disseminated disease. We present the case of a 29-year-old liver transplant recipient who developed aggressive PM, confirmed through bronchoalveolar lavage. Treatment involved liposomal amphotericin B, followed by surgical debridement and right pneumonectomy. Despite these interventions, persistent infection required an Eloesser flap procedure for enhanced drainage. This case underscores the importance of early diagnosis and aggressive, multidisciplinary treatment approaches, including surgery and antifungal therapy, to improve outcomes in immunocompromised patients facing this life-threatening condition.

## Introduction

Pulmonary mucormycosis (PM) is a fungal infection caused by fungi from the Mucorales order, primarily affecting immunocompromised individuals, such as those with diabetes mellitus, hematologic malignancies, and recipients of solid organ transplants [1]. The pathogenicity of Mucor lies in its ability to rapidly proliferate and invade blood vessels, leading to angioinvasion, thrombosis, and tissue necrosis [2]. This early angioinvasion enables the fungus to spread quickly, resulting in severe damage to the lungs and, in many cases, dissemination to other organs. The rapid progression of the infection makes timely diagnosis and intervention crucial for patient survival, yet this remains a significant clinical challenge [2].

PM is associated with alarmingly high mortality rates ranging from 50% to 80% for cases confined to the lungs and nearly 100% for disseminated disease [1]. The disease is particularly concerning in solid organ transplant recipients, where the use of immunosuppressive therapy increases susceptibility [3]. In fact, mucormycosis is estimated to occur in approximately 2% of solid organ transplant patients, making this group particularly vulnerable [3]. Other risk factors for mucormycosis include prolonged corticosteroid use, neutropenia, and iron overload, all of which impair the body's immune defenses [1].

The clinical presentation of PM can be subtle and nonspecific, often mimicking other respiratory infections, such as bacterial pneumonia, tuberculosis, or invasive aspergillosis [2]. This diagnostic ambiguity is compounded by the lack of highly sensitive and specific non-invasive diagnostic tests, with definitive diagnoses relying on invasive procedures such as bronchoalveolar lavage (BAL) or lung biopsy [4]. Imaging studies such as CT scans can suggest mucormycosis through findings such as pulmonary nodules, cavitation, or consolidation, but these features are not pathognomonic [5]. Common symptoms include cough, hemoptysis, chest pain, dyspnea, and fever, which are easily mistaken for other pulmonary conditions [5]. Consequently, diagnosis is frequently delayed, often until the infection has progressed to an advanced stage.

The management of PM requires a multimodal approach. Early and aggressive treatment is vital for improving prognosis, yet therapeutic success is hindered by the infection's rapid progression and the limitations of available antifungal agents. Liposomal amphotericin B remains the first line of treatment due to its efficacy and reduced toxicity compared to conventional formulations [6]. However, antifungal therapy alone is often insufficient, particularly in cases where significant tissue necrosis has occurred [2]. Surgical resection of the infected tissue, such as pneumonectomy or lobectomy, is frequently necessary to control the spread of the infection [7]. This approach, while aggressive, has been shown to improve outcomes in select patients, particularly those who are able to tolerate the physiological demands of surgery [8].

Despite these interventions, the prognosis for patients with PM remains poor, especially in cases where the diagnosis is delayed or the infection has disseminated [1]. Immunocompromised patients, particularly those who have undergone solid organ transplantation, require heightened clinical vigilance and prompt diagnostic evaluation to detect mucormycosis at an early stage. The importance of a multidisciplinary approach, involving infectious disease specialists, pulmonologists, surgeons, and radiologists, cannot be overstated in the successful management of this life-threatening infection.

This case report aims to highlight the diagnostic and therapeutic challenges of managing PM in an immunocompromised patient, emphasizing the critical need for early diagnosis, timely surgical intervention, and appropriate antifungal therapy. The report also underscores the necessity of a coordinated, multidisciplinary approach to improving patient outcomes, particularly in high-risk populations such as solid organ transplant recipients.

## Case presentation

A 29-year-old patient underwent liver transplantation due to alcohol-induced liver damage and was subsequently administered the immunosuppressant tacrolimus to prevent graft rejection. On hospital day three, the patient exhibited signs of rapidly progressing pneumonia. Laboratory results showed a white blood cell count of 17.74 x 10^3^/µL, hemoglobin of 10.3 g/dL, and a platelet count of 54 x 10^3^/µL. Chest X-rays revealed opacities throughout the right lung lobe, which raised concerns for pulmonary mucormycosis, subsequently confirmed by bronchoalveolar lavage, which identified irregular, non-septate hyphae with wide-angle branching. Treatment included liposomal amphotericin B, posaconazole, micafungin, and hydrocortisone. Due to mucormycosis's aggressive nature, surgical debridement of all affected areas was necessary. The patient underwent an immediate pneumonectomy via lateral thoracotomy due to extensive disease infiltration. The removed lung was dense and edematous. Draining tubes were placed to drain the remaining mucor. On hospital day five, the patient exhibited complications from residual mucormycosis. The cardiothoracic team intervened with an Eloesser flap procedure to manage the persistent mucor infection. The physicians’ primary aims of this thoracostomy flap were to allow passive drainage of the infected pleural cavity and to create a one-way valve facilitating fluid efflux from the thoracic area. Table 1 presents the laboratory investigation results, and Table 2 presents the timeline of the event.

**Table 1:** Laboratory investigation results of a 29-year-old liver transplant patient with pulmonary mucormycosis. This table includes key hematological parameters, such as white blood cell count, hemoglobin levels, and platelet count, with reference ranges for comparison. The elevated white blood cell count and low platelet count reflect the patient’s inflammatory response and underlying hematological abnormalities.

Parameter	Obtained Value	Reference Range
White Blood Cell Count	17.74 x 10^3^/µL	4.0-11.0 x 10^3^/µL
Hemoglobin	10.3 g/dL	13.5-17.5 g/dL (males), 12.0-15.5 g/dL (females)
Platelet Count	54 x 10^3^/µL	150-450 x 10^3^/µL

**Table 2:** Timeline.

Timeframe	Event
Hospital Day 1	Liver Transplant: Patient admitted and liver transplant performed. Postoperative Care: Transfer to the intensive care unit (ICU) for monitoring.
Hospital Day 3	Postoperative: Symptoms indicating pulmonary infection appear. Diagnosis: Pulmonary mucormycosis identified via bronchoalveolar lavage. Surgical intervention: Right pneumonectomy and debridement were conducted.
Hospital Day 5	Post-Surgery: Persistence of mucormycosis, requiring additional management. Further Procedure: Eloesser flap procedure executed for ongoing infection control.

**Figure 1:**
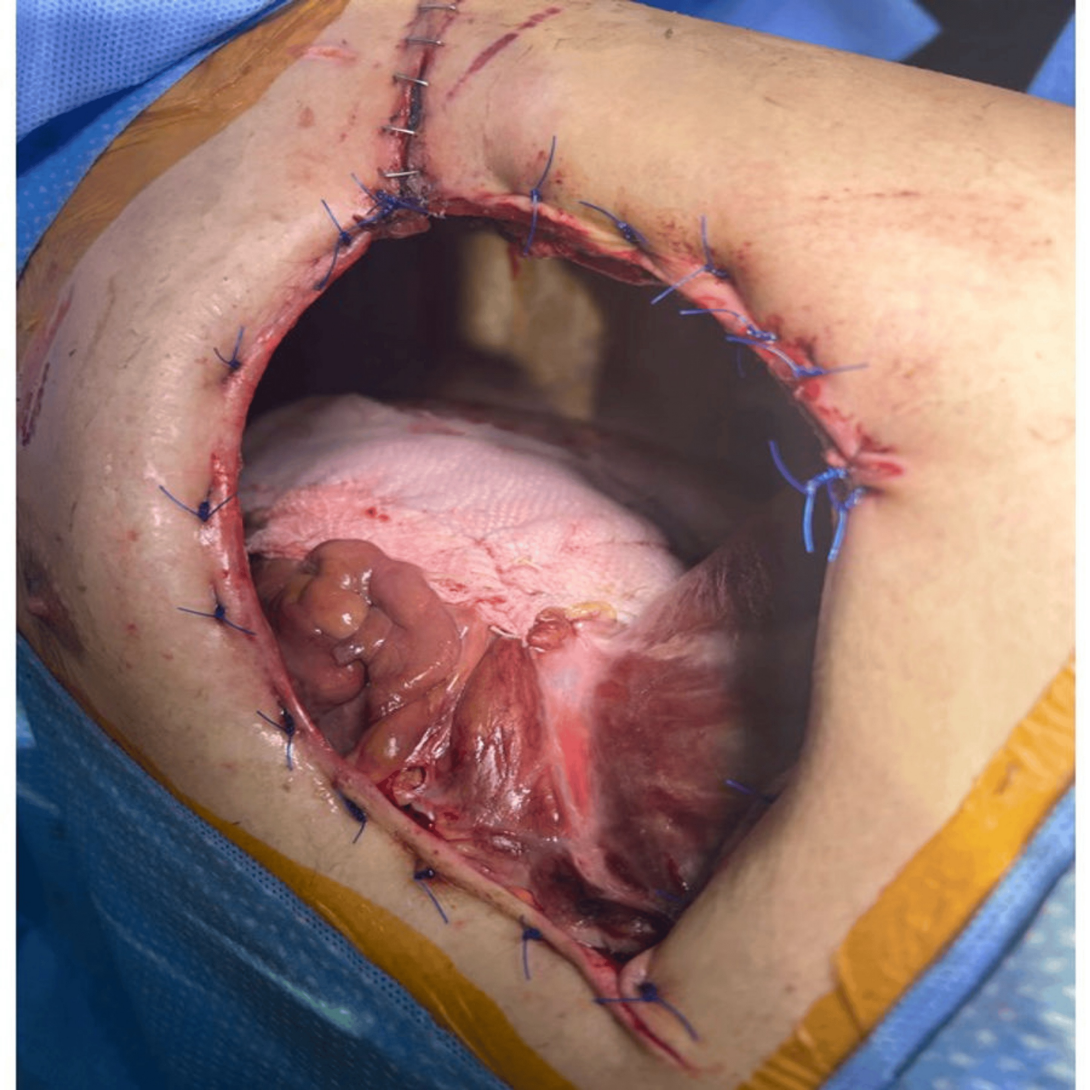
Right hemithorax status after the Eloesser flap procedure.

Figure 1 presents the right hemithorax status - post-Eloesser flap procedure.

## Discussion

In immunocompromised patients, such as those on tacrolimus, the risk of developing PM is significantly heightened due to neutropenia, which results from the drug’s inhibition of hematopoiesis and induction of apoptosis via lymphocyte-secreted cytokines [9]. Neutropenia, along with impaired innate immune responses, leaves these patients particularly vulnerable to the rapid progression of mucormycosis [9]. This case highlights the need for a high index of suspicion for PM in transplant recipients presenting with respiratory symptoms, especially in those with neutropenia or prolonged immunosuppression.

PM remains a formidable challenge in clinical practice, particularly among immunocompromised populations such as post-transplant patients. The standards for diagnosis of PM include sputum culture, bronchoalveolar lavage, and biopsy [10]. These methods prove useful, yet come at the expense of time, which can allow further disease involvement if not properly identified.

One of the key challenges in managing PM is the rate with which the infection progresses, as it spreads one inch per hour [11]. Mucorales fungi invade blood vessels early in infection, causing tissue necrosis, thrombosis, and potentially severe disease dissemination, which greatly increases mortality and makes early intervention critical [2]. In this case, the patient presented with an advanced form of PM that required immediate surgical intervention. Surgical debridement and pneumonectomy were performed to remove necrotic lung tissue and prevent further dissemination of the infection. However, despite the aggressive nature of the initial intervention, the patient’s condition continued to deteriorate, necessitating further surgical measures, including an Eloesser flap procedure to enhance drainage and prevent additional spread of the infection.

The Eloesser flap, which involves the creation of an open thoracic window for ongoing drainage, is typically reserved for severe cases where conventional interventions have failed [12]. Its use in this case highlights the importance of adopting a flexible, patient-tailored approach when managing PM. While the procedure carries significant risks, its ability to promote drainage and limit further necrosis was crucial in this patient's care. The case illustrates that, in advanced mucormycosis, surgical interventions must not only be prompt but also adaptable to the patient’s evolving clinical condition.

This case also emphasizes the importance of a multidisciplinary approach in managing PM. In addition to antifungal therapy with liposomal amphotericin B, the involvement of surgical teams was essential for controlling the infection. Studies suggest that combined medical and surgical treatment strategies significantly improve survival rates in patients with PM, with reported survival rates of 69% for combined therapy compared to just 31% for medical therapy alone [13]. Early surgical consultation, particularly in intensive care unit (ICU) settings, is therefore recommended for any patient with suspected PM, especially those with immunosuppressive conditions or neutropenia [14]. In this case, early engagement with cardiothoracic surgeons allowed for prompt surgical debridement, which played a critical role in managing the infection.

Furthermore, this case demonstrates the value of close collaboration among ICU physicians, radiologists, surgeons, and infectious disease specialists. Such a multidisciplinary approach ensures swift identification and management of PM, improving the likelihood of a favorable outcome. Radiologists play a pivotal role in detecting early signs of fungal invasion through imaging techniques, while infectious disease specialists guide the antifungal regimen. The timely involvement of cardiothoracic surgeons, especially when there is radiologic evidence of angioinvasion or necrosis, is paramount to preventing disease progression and reducing mortality.

## Conclusions

The aggressive management of pulmonary mucormycosis in post-transplant patients requires a prompt and multidisciplinary approach. Early detection, combined with swift antifungal therapy and surgical intervention, is essential to improving survival outcomes, as demonstrated by this case. The integration of medical and surgical treatments, such as the use of an Eloesser flap, highlights the importance of coordinated care in preventing disease progression and mitigating the high mortality associated with this infection in immunocompromised patients.
